# THE IMPACT OF AN INTERPROFESSIONAL SIMULATION ON TEAM COMMUNICATION AT THE END OF LIFE

**DOI:** 10.1093/geroni/igz038.541

**Published:** 2019-11-08

**Authors:** Anne Halli-Tierney, Megan E Lippe, Alexandra Stanley, Allison Ricamato, Robert E McKinney

**Affiliations:** University of Alabama, Tuscaloosa, Alabama, United States

## Abstract

It is increasingly important that healthcare professionals understand how to care for patients at the end of life, especially as 75% of hospital deaths occur in the geriatric population. Many new healthcare profession graduates feel under-prepared to provide end-of-life care. Healthcare education programs must provide education on interprofessional communication and end-of-life care. This study explored the effects of an interprofessional end-of-life simulation on medical, social work, and nursing students’ communication skills. Across three simulation phases, teams were required to communicate with the patient (high-fidelity manikin), family members (scripted actors), and team members about treatment option, change in patient’s condition, and withdrawal of life-sustaining measures. A total of 16 teams participated in the simulation, with each team comprised of nursing students (n=7-8), social work students (n=1), and medical students or residents (n=1). Team communication skills were assessed using the Gap Kalamazoo Communication Skills Assessment Form. The average communication score for teams reflected fair to good communication (M=28.81, SD=5.55). The best communication domains were “Shares information” (n=8), “Communicates accurate information” (n=6), and “Builds a relationship” (n=5). The worst domains were “Demonstrates empathy” (n=7), “Provides closure” (n=6), and “Builds a relationship” (n=5). This simulation provides an interactive educational mechanism by which to educate interprofessional healthcare students on communication and care of patients at the end of life.

